# Supporting quality care for ESRD patients: the social worker can help address barriers to advance care planning

**DOI:** 10.1186/s12882-020-01720-0

**Published:** 2020-02-19

**Authors:** Charles R. Senteio, Mary Beth Callahan

**Affiliations:** 1 School of Communication and Information, Rutgers University, 4 Huntington Street, New Brunswick, NJ 08901 USA; 2 Dallas Nephrology Associates, 411 North Washington Street, Suite #7000, Dallas, TX 75246 USA

**Keywords:** Advance care planning, End-of-life care, ERD quality care

## Abstract

**Background:**

Advance Care Planning (ACP) is essential for preparation for end-of-life. It is a means through which patients clarify their treatment wishes. ACP is a patient-centered, dynamic process involving patients, their families, and caregivers. It is designed to 1) clarify goals of care, 2) increase patient agency over their care and treatments, and 3) help prepare for death. ACP is an active process; the end-stage renal disease (ESRD) illness trajectory creates health circumstances that necessitate that caregivers assess and nurture patient readiness for ACP discussions. Effective ACP enhances patient engagement and quality of life resulting in better quality of care.

**Main body:**

Despite these benefits, ACP is not consistently completed. Clinical, technical, and social barriers result in key challenges to quality care. First, ACP requires caregivers to have end-of-life conversations that they lack the training to perform and often find difficult. Second, electronic health record (EHR) tools do not enable the efficient exchange of requisite psychosocial information such as treatment burden, patient preferences, health beliefs, priorities, and understanding of prognosis. This results in a lack of information available to enable patients and their families to understand the impact of illness and treatment options. Third, culture plays a vital role in end-of-life conversations. Social barriers include circumstances when a patient’s cultural beliefs or value system conflicts with the caregiver’s beliefs. Caregivers describe this disconnect as a key barrier to ACP. Consistent ACP is integral to quality patient-centered care and social workers’ training and clinical roles uniquely position them to support ACP.

**Conclusion:**

In this debate, we detail the known barriers to completing ACP for ESRD patients, and we describe its benefits. We detail how social workers, in particular, can support health outcomes by promoting the health information exchange that occurs during these sensitive conversations with patients, their family, and care team members. We aim to inform clinical social workers of this opportunity to enhance quality care by engaging in ACP. We describe research to help further elucidate barriers, and how researchers and caregivers can design and deliver interventions that support ACP to address this persistent challenge to quality end-of-life care.

## Key points

- ACP is a process involving ESRD patients, their families, and caregivers to clarify goals, enable shared decision-making, and prepare for death.
- Clinical, technical, and social factors present barriers to ACP.
- Social workers’ skills and role on caregiver team enable them to support ACP.

## Background

*Wherever your life ends, it is all there. The utility of living consists not in the length of days, but in the use of time; a man may have lived long, and yet lived but a little*.

Michel Eyquem de Montaigne, 1533–1592

There is an opportunity for social workers to help facilitate quality care by performing advance care planning (ACP), which requires the collection and use of psychosocial information. The ACP process is a critical aspect of a patient-centered approach to care, and it represents an important, necessary step to align chronic kidney disease (CKD) patients’ treatment plans to their preferences, values, and goals [1]. ACP provides patients and their families a conduit to clarify their treatment wishes [2, 3].

ACP is a patient-centered, dynamic process involving patients, their families, and caregivers. The ACP process is designed to 1) clarify goals of care, 2) increase patient agency over their medical care and treatment, and 3) help prepare for death. ACP is an active process because the CKD illness trajectory creates health circumstances that necessitate that clinicians assess and nurture patient readiness to participate in ACP discussions. This requires that conversations are informed by shifting psychosocial information that helps describe the patient’s situation. Thus, ACP must be revisited at appropriate times, driven by intervals based on both clinical and psychosocial considerations [4, 5].

ACP and advanced directives are not synonymous. ACP is distinct from simply documenting the existence and content of advanced directives. Advanced directives are documents that describe the patients’ wishes concerning medical decisions in the event that they are not able to make those decisions themselves, in essence, if they lose decision-making capabilities [6]. While ACP may result in the *creation* of an advanced directive, ACP requires the exchange of personal, sensitive psychosocial information that must be periodically revisited.

Psychosocial information is indispensable to the ACP process. Psychosocial information includes individual (e.g., individual perceptions and trust) and structural (e.g., level of social support and cultural tradition) information fundamental to ACP [7–9]. Requisite information from patients and family members who comprise their support system includes intimate, personal information which informs the key quality of life considerations. This personal information can include values, beliefs, and priorities regarding: what medical/clinical treatments they wish to undergo, and for what length of time, what medical/clinical conditions they want to be treated and what outcomes (i.e., disabilities) they are willing to live with [6].

In limited study populations, nephrologists who are prompted to identify patients who might benefit from ACP demonstrate its efficacy via enhanced documentation of patient preferences for limits on life-sustaining treatment [10, 11]. ACP is particularly imperative for older adults, as they represent the fastest-growing population starting dialysis [12]; 65 is the current median age of end-stage renal disease (ESRD) patients who begin dialysis, and 10% of all patients above 75 who start dialysis die within *3 months* [13] and 41% die within 1 year [14].

Access to ACP can enhance the quality of life through patient knowledge that their wishes are known and will be followed. In some cases, empowerment through disease process knowledge can extend life through increased engagement, especially among older adult patients. In one study (*N* = 122), one-third of patients whose ACP resulted in the choice to engage in palliative care and stop treatment survived 12 months beyond the time when dialysis would have been indicated, and over half (58%) experienced stable or improved quality of life [11, 15]. Despite this empirical support for the benefits of ACP, considerable research describes that patients do not consistently receive treatment that considers their preferences and goals [16–20]. ESRD patients desire information concerning their prognosis, and patients who have access to this information tend to prefer, and hence choose, less aggressive treatment options [21]. But few dialysis patients report having even limited conversations about their wishes concerning end-of-life; ESRD patients report that treatment options that include discontinuing dialysis are *rarely even discussed* [22].

Various clinical, technical, and social barriers to psychosocial information exchange have been described. Clinical barriers include fear of taking away hope from patients and clinician bias towards aggressive treatment [10, 20]. Technological barriers include the inability to collect and use psychosocial information, specifically information that includes patients’ goals and values [23]. Current technology capabilities i.e., electronic health record (EHR), impede the collection and use of psychosocial information across various ambulatory care settings [24]. However, we acknowledge that EHR capabilities enable data collection and analysis that support the creation and evaluation of models that calculate the risk of disease progression, important to facilitate an ACP discussion. Since physical data from CKD patients is regularly recorded in clinical practice, various models have been built which attempt to identify factors which predict kidney disease progression [25–30]. For example, one model that used longitudinal laboratory test results and clinical documentation predicted CKD progression from stage III to stage IV more accurately than other models that do not account for these factors [31]. Social barriers include patient belief systems which may conflict with caregivers’ values [19]. We also acknowledge that legal considerations are occasionally cited as barriers; however, we concur with the prevailing view that since advance directives tend to *not dictate control* over medical procedures as do, for example, wills over property, in practice the existence (or lack thereof) of ACP does little to prevent legal disputes over medical care [32].

Social workers’ role and training position them well to help facilitate ACP and enable the requisite psychosocial information exchange. Further, social workers can support the design of interventions to address the persistent issue of lack of consistent ACP for ESRD patients.

### ACP and patient-centered care

The Coalition for Supportive Care for Kidney Patients (CSCKP), formerly the Kidney End-of-Life Coalition, states that a patient-centered approach to care that includes ACP can result in better quality of care for ESRD patients as defined by the National Quality Strategy *Three Aims*: 1) better care for the individual, 2) better health for populations, and 3) reduced cost of care [1]. As described in the National Quality Strategy, the United States Centers for Medicare and Medicaid Services (CMS) holds practices accountable for quality measures that help define value-based care, and *patient engagement and experience* is one of the three measures specifically pertinent for ACP; the other two are clinical care metrics and care coordination [33]. ACP discussions include patient and family wishes, so this is a particularly important indicator of patient engagement which can have a dramatic influence on the plan of care [34]. For example, for practices in which caregivers work closely with patients to understand their treatment preferences, patients choose lower-cost and less-intense treatment [35–37]. The considerable ESRD treatment burden – spiritual, psychosocial, and physical – further illuminates the need to include ACP in the care management of ESRD patients [38].

#### Information exchange required for ACP

ACP has evolved from simply designating a health care proxy (i.e., surrogate) or instructions (i.e., living will, advanced directive) which emerge from a singular conversation between physician and patient – to a process of ongoing psychosocial information exchange, essential for delivering patient-centered, quality care and honoring the patients’ values. The “living will” was first conceived in 1969 as a legal document, a trust that establishes the physician as the trustee and patient as the beneficiary [32]. Today, significant health information exchange is necessary in order to fulfill key goals of ACP: clarifying treatment goals, supporting patient agency over their care, and preparing for death and bereavement [4]. Since patient priorities, wishes, and goals may change over time, especially as health circumstances change over CKD progression, this health information exchange must be revised periodically. In fact, the CMS mandates patient care plan assessments that include serious illness conversations at minimum annually, but also according to specific clinical events or circumstances such as: upon admission to a dialysis unit, after 3 months on dialysis, or after prolonged hospital admission [39]. Non-time based triggers to prompt serious illness conversations have been detailed for patients prior to dialysis (e.g., transplant referral, repeated or extended hospitalizations, changes in functionality, falling albumin, or weight loss) and after dialysis (e.g., access procedures, sentinel events such as falls and sudden appetite change) [40]. A reciprocal exchange of sensitive health information is required for these conversations, necessary to fulfill the following key principles of ACP [6, 41, 42]:

- Understand the patients’ priorities, what is important to them and why.
- Comprehend what the patient wants or does not want to be treated.
- Appreciate what clinical treatments the patient wants or does not want.
- Discover what outcomes the patient wants or does not want to live with. Include specific symptoms that are important to the patient. Know what general quality of life issues are important to the patient.
- Know who the patient wants to speak to their wishes in the event that the patient cannot make their own decisions regarding care.
- Grasp to what degree the patients’ engaged family members are aware of their preferences.

### Known barriers to ACP: clinical, technical, and social

ACP is not consistently completed due to clinical, technical, and social barriers that prevent requisite psychosocial information from being collected or used. Clinical barriers include lack of training and practice policies that may dissuade less aggressive treatment which more informed patients may select. Technology barriers include current tools i.e., EHR, that are ill-equipped to collect the psychosocial information needed to inform ACP. Social barriers include cultural values that may conflict with caregivers’ values.

Clinical barriers to access and use of psychosocial information required to complete ACP are grouped in three practice areas: 1) education/training, 2) implementation, and 3) policy. First, caregivers are not adequately trained to address the deleterious perceptions that ACP is *merely* “end-of-life” care and the general assumption that dialysis is categorically consistent with the goals of ESRD patients [21, 43]. Second, ACP programs are not developed and implemented because practices lack caregivers trained to implement ACP, resulting in an inability to identify patients appropriate for referral. Last, policy barriers include the need for more research on the clinical and financial effects of ACP which include consideration of reimbursement policies and regulatory obstacles [44]. As a consequence of these barriers, ACP has not been widely implemented despite research and policy leaders acknowledging that improved access to ACP holds a considerable opportunity to improve *both* clinical outcomes [45] and financial value [46]. There is a vital need to investigate the entire effect of ACP; lessons learned should be used to refine, measure, and expand its capacity.

#### Clinical education / training

In large surveys, nephrologists report that they are not prepared to support patients in end-of-life decisions, and nephrology residents report insufficient palliative care education necessary to conduct ACP [47]. For example, in one of the largest surveys of its kind, a 2006 multi-national survey of nephrologists across the United States found that only 39% of nephrologists felt “very well prepared” to support end-of-life decision making with patients [48]. Less than 5% of nephrology care teams across the United States report having the skills *across their interdisciplinary teams* to effectively conduct ACP [5]. The literature indicates that uncertainty concerning prognosis is a key missing aspect of training in residency, fellowships, and continuing education [21]. In fact, rapidly evolving predictive models that indicate functional status are being developed to help prompt a reassessment of a patient’s wishes or a change in prognosis [49, 50]. Despite these important, developing capabilities, the literature is virtually silent in describing prompts for other members of the care team, such as nurses and social workers [51]. However, nephrology nurses describe difficulties in performing, or in advocating for end-of-life care due to other clinical demands, and competing priorities among themselves and physicians and practice leadership [52]. Further, for caregivers ACP discussions may represent a discussion about failure rather than fulfilling the obligation to engage with the patient to share in decision-making. Although caregiver perceptions of ACP vary according to patient conditions and training, the discomfort associated with conversations with patients about death and dying, and apprehension that doing so will create anxiety, have been described as barriers to initiating ACP [53].

#### Clinical implementation

The Renal Physicians Association’s clinical practice guidelines address ACP. The guidelines detail specific questions to ask patients to help evaluate their wishes [54]. The guidelines also include the commonly named “surprise question” physicians can ask themselves to help inform ACP: “Would I be surprised if this patient died in the next year?” However, the literature lacks descriptions of how ACP may be conducted and specific steps to implement it [10, 11].

Despite considerable advances in clinical decision support tools that have increased the quality of care and patient safety, the development of these tools has not yet reached support for predictive ESRD prognosis [55]. In fact, among dialysis caregivers and administrators, the lack of comprehensive decision support and guidelines to support decision making for ESRD patients is a principal barrier to end-of-life discussions across CKD practices. For example, in a large survey (*N* = 487) of ESRD caregivers – nephrologists, nurse practitioners, nurses, social workers, and dialysis administrators – across dialysis centers respondents, 40% of whom were social workers, indicated that they were unaware of *any* validated prognostic model or clinical practice guidelines to support end-of-life decisions [56]. There are few examples of clinical decision support tools designed for clinical practice that attempt to identify dialysis patients at the *highest risk* of death; however, none have been validated and used in routine clinical practice [57]. For example, the Charlson Comorbidity Index (CCI) is a tool to estimate mortality risk from comorbid disease [58]. In limited study populations, the CCI has been investigated to inform clinical decision support tools for six-month survival for dialysis patients [59–62].

However, other than number and frequency of hospitalizations; there are no “milestone” guidelines in systematic, routine clinical use so that caregivers can evaluate parameters to help inform care goals, which may include withdrawal from dialysis [47]. This results in clinical environments in which caregivers are reluctant to have discussions which they believe will *elicit fear* and *take away hope* from ESRD patients and their families [63].

#### Policy – financial incentives/reimbursement

In 2011 the CMS introduced bundled payment reform in conjunction with the Quality Incentive Program (QIP) in an effort to enhance efficiency for ESRD care. This policy shift has resulted in practices responding to QIP stipulations, which include up to 2% reimbursement penalties if selected quality indicators are not met [64]. Effective January 2016 the CMS instituted reimbursement for *voluntary* ACP under the Medicare Physician Fee Schedule (MPFS) [65]. Despite these changes, end-of-life care has been virtually unchanged in large part because performance measures are associated with clinical goals not related to ACP [66]. For example, performance metrics include dialysis dose, hemoglobin levels, and maximizing arteriovenous fistula while minimizing central venous catheter use [21]. In fact, these incentives may run *contrary* to ACP in cases where patients subsequently elect for less-aggressive treatments. Treatment goals that result from ACP may be inconsistent with these disease-focused incentives. Incentivized metrics may create incongruent priorities, especially among caregivers who may wish to advocate for patient-centered care, yet who may also have a personal or financial interest in volume and/or reported clinical metrics [21]. Indeed, in limited studies, the literature supports that financial incentives may dissuade caregivers from having ACP discussions that may result in less-aggressive treatment [16]. Despite these circumstances, alternative financial models are in place that show promise to better align ESRD incentives with patient-centered care. For example, a comprehensive ESRD care model enables organizations to incorporate palliative and hospice care into “regular” ESRD care for patients with end-of-life care needs [5].

### Technical barriers

Clinicians across specialties acknowledge the need for better collection and use of psychosocial information, primarily due to limited capabilities to do so given current capabilities of EHR tools [24, 67]. In fact, the literature describes recommendations that current EHR tools should be enhanced to include a patient care plan, which aims to promote patient engagement and collaboration with caregivers across care teams and the healthcare system [68]. Specifically, the EHR should include capabilities to access and use psychosocial information, which of course the patient provides. Recommendations include enhancing EHR capabilities to document and use psychosocial information such as a patients’ emotional health and stressors, as this information is key to help inform various clinical decisions [69, 70]. For instance, a caregiver could capture how a patient’s cultural and/or spiritual beliefs influence their views concerning end-of-life planning. A national survey of 1000 primary care physicians identified that access to psychosocial information is critical to influencing patient health outcomes. A large majority of respondents (85%) indicated that *they do not feel confident* in currently available tools to assess and address patients’ social, cultural, and emotional needs [71]. Caregivers must have consistent access to psychosocial information in order to understand patients’ values, goals, and care needs as its collection and use are necessary to perform ACP. The caregiver must use psychosocial information to inform critical care decisions concerning the plan of care, especially decisions which may result from ACP.

### Social barriers

Caregivers across various clinical roles express concerns for circumstances when the patient’s cultural beliefs and values may be discordant with the caregivers’ belief systems [19]. In fact, nephrology nurses describe conflicting belief systems among key barriers to performing ACP [51, 72]. The literature describes the vital role that culture plays in conversations about end-of-life. Culture influences the role of family and community relationships, spirituality, worldview, and it even shapes perceptions of appropriate communication because cultural norms influence the very role of language [73]. Consequently, international guidelines call for increased research on how to incorporate culturally responsive language and communication into ACP [74]. Emerging literature describes the culture-specific preferences and values which have a profound impact on ACP discussions. For example, when compared to other ethnic groups, Latinos on dialysis are less likely to have an advance directive and they are less likely to die with hospice services [74]. African American patients with kidney disease tend to have discordant beliefs about the meaning of death or illness when compared to their caregivers. Also, African American patients, in general, tend to hold higher levels of mistrust for health care delivery systems, which exacerbate the difficulty of end-of-life communication and decisions [75]. Consequently, when compared to White patients, African American patients are less likely to report conversations about end-of-life preferences and knowledge of treatment options (e.g., hospice) [76]. These insights reflect that much of the research concerning cultural-specific preferences and values stem from North America and Canada, where caregivers and patients tend to have more experience with ACP. However, international cultural perspectives are emerging. In one study of Irish patients receiving hemodialysis, the majority were comfortable talking about end-of-life and death, but family members were most comfortable with discussions concerning prolonging life, specifically preferring medical interventions to prolong life [77]. In another study of dialysis patients in Italy, a significant proportion of patients preferred not to be involved with end-of-life decisions, and older adult patients preferred to continue dialysis despite poor quality of life indicators or poor prognosis [78].

As a result of clinical, technical, and social barriers, ESRD patients experience substantial unmet ACP needs. There is a compelling need for enhanced psychosocial information exchange that enables education on the disease and its progression and facilitates support for supportive dialogue crucial to ACP [79]. ESRD patients continue to report needing information concerning their illness and prognosis [80], and across studies, ESRD patients report that their care team rarely discusses ACP goals [5, 41].

### Social work’s role in ACP

Quality of care for ESRD patients includes ACP, which requires a partnership between patients, their families, and caregivers [81]. Due to high mortality, ACP is particularly important for late-stage CKD and dialysis patients. One in five dialysis patients die each year [82], and more stage 4 CKD patients die prior to developing ESRD (8.0 per 100 patient-years) than those who develop ESRD (7.7 per 100 patient-years) [83]. Despite its suitability, ACP has not been widely adopted across nephrology practices due to obstacles to psychosocial information exchange based on clinical, technical, and social barriers [44]. The nephrology social worker’s role continues to evolve to include key assessment and patient-support tasks for interventions focused on patient health behavior (e.g., medication recommendations, dietary and fluid guidelines) [84]. In fact, the CMS mandates a master’s level social worker in all dialysis centers to help assess and support emotional factors known to influence health behavior. Although social workers remain largely excluded from CKD management, they are consistently valued members of palliative care teams. Further, leading (for profit) dialysis providers support psychosocial information exchange that can help assess and address barriers to recommended health behavior [85, 86].

In fact, recent research offers emerging insights on how to design and evaluate ACP interventions. This research confirms that nephrology social workers are *already* playing key roles in designing and executing interventions aimed to enhance the quality of care [87]. Tools are being developed to help improve information exchange concerning prognosis and treatment options, and patients’ values and beliefs [76]. Key lessons learned from these ACP interventions include: a) employing practice-level efforts focused on patient preferences and values, b) using interdisciplinary care teams, and c) empathic listening and focus on patient engagement at the very onset and throughout ACP.

As described, clinical barriers concerning conflicting motivations are quite daunting. However, examining the emerging literature describing practice level ACP interventions is informative, and doing so helps make the case for practice level ACP. The rationale should include identifying existing policies and procedures, whether explicit or tacit, that presumes a set plan of care. The impetus for these organizational shifts is grounded in patient engagement and considering their needs, values, and preferences [5]. Few caregivers and administrators will refute the importance of shared priorities concerning patient-centered care, which can begin with patients who have already completed advanced directives; incorporating them into a more comprehensive ACP process can instill confidence that patients’ stated wishes are understood and will be honored [88, 89].

Constituting interdisciplinary teams is critical in order to address the innumerable information needs and concerns that can arise during ACP. Patients have diverse needs and preferences, which can shift over time, so an interdisciplinary team is necessary in order to, for example, support patients’ various biopsychosocial and spiritual needs [14]. The training and experience that social workers bring to an interdisciplinary team make them critical to addressing the clinical, technical, and social barriers to psychosocial information collection and use as it pertains to ACP.

Empathic listening during the ACP conversations, and in the discussions leading up to them, is critical to help facilitate ACP because it facilitates understanding of the patients’ values and wishes [42, 90]. This is important to establish patient buy-in, which is achieved by simultaneously affirming a genuine sense of agency while identifying treatment options associated with ACP. The patients who engage in these discussions tend to require more information, so the focus needs to remain on the patient and how the disease progression and treatment options may affect their lives according to their own values and relationships. Social worker training includes motivational interviewing (MI). a skills-based approach to helping individuals change behavior [91]. MI may be particularly suited to ACP because it requires adjustment based on the patient’s readiness to engage in requisite conversations and reflection. In fact, MI has been used in ESRD care to assist with fluid management, and help reduce depression and anxiety [72]. ACP cannot be completed through pre-determined routinized, specific steps. MI enables flexibility to adjust to the patient’s readiness level and is sensitive to identifying when a patient may be receptive (i.e., ready) to have an ACP conversation [92]. MI also can help adjust to the diversity of cultural values, and MI has shown efficacy in improving medication adherence for African American patients [72]. Given their proximity to patients, the nature of the information disclosure that already occurs, and their training in MI, social workers can help align practice decisions resulting from ACP with patient perceptions and goals, values, and preferences.

Since patient engagement is such a crucial dimension, future ACP interventions should leverage lessons learned from studies aimed at enhancing ESRD patient engagement, specifically concerning living donor transplantation and the importance of training caregivers using ‘model conversations’ that employ both print i.e., booklets and technology, i.e., short videos, featuring ethnically diverse patients and family members [93].

## Conclusion

In this review, we outline the clinical, technical, and social barriers to collecting and using the psychosocial information required to perform ACP. Our goal is to equip nephrology care teams with information concerning the benefits of ACP, insights on the known barriers to ACP, and strategies to address them. Nephrology social workers are uniquely positioned to help enhance quality via improved patient engagement. Designing and executing ACP interventions that help to identify ACP perceptions and barriers is an important initial step, and social workers should be at the forefront of implementing ACP in their advocacy for patients and their families.

## Data Availability

Not applicable.

## Abbreviations

ACP: Advance care planning
CCI: Charlson Comorbidity Index
CKD: Chronic kidney disease
CMS: United States Centers for Medicare and Medicaid Services
EHR: Electronic health record
ESRD: End-stage renal disease
MI: Motivational interviewing
QIP: Quality Incentive Program
